# Diabetic foot osteomyelitis: pathological microenvironment and matching tailored treatment strategies

**DOI:** 10.3389/fcell.2026.1794273

**Published:** 2026-02-26

**Authors:** Shi-jiu Yin, Jia Li, Yi Ren, Hai Yang, Ya-xing Li, Hui Zhang

**Affiliations:** 1 Department of Orthopedic Surgery and Orthopedic Research Institute, West China Hospital, Sichuan University, Chengdu, Sichuan, China; 2 Department of Orthopaedic Surgery, Shangjin Nanfu Hospital, Chengdu, Sichuan, China

**Keywords:** bone tissue engineering materials, diabetic foot osteomyelitis (DFO), multi-target therapy, pathological microenvironments, tailored treatment strategies

## Abstract

Diabetic foot osteomyelitis (DFO) often manifests as persistent, non-healing infection with progressive bone destruction. Poor glycemic control and concomitant peripheral vascular and neuropathic injury are key drivers. Effective clinical solutions remain limited. Current management typically involves debridement of infected and necrotic tissues, local or systemic antibiotics, and bone/soft-tissue reconstruction. However, impaired local circulation makes it difficult to sustain therapeutic antibiotic levels at the lesion site. Recurrence is therefore common. Bone regeneration is also hard to achieve, which prolongs the overall course and results in repeated procedures, long recovery cycles, and high costs. To overcome these limitations, we propose a microenvironment-matched strategy as a practical direction for DFO therapy. The DFO niche is characterized by bacterial persistence and recurrent infection, severe oxidative stress and chronic inflammation, immunometabolic dysregulation, and microvascular plus neural injury that suppress osteogenesis. These constraints converge on three intertwined therapeutic targets: infection, inflammation, and bone defects. Treatment should thus be precise, sequential, and coordinated across targets, rather than relying on isolated interventions. This review systematically summarizes advances in multitarget antibacterial approaches, anti-inflammatory and immunometabolic modulation, and multifunctional biomaterial platforms that integrate angiogenesis, neurorestoration, and osteogenic regeneration. We further highlight microenvironment-responsive, integrated strategies that optimize drug dosing and release timing, aiming to improve the durability of infection control and the quality of bone reconstruction. Ultimately, we provide researchers with testable material design and synthesis logic, and offer clinicians new therapeutic paradigms and stage-adaptive, precision care pathways.

## 1 Introduction

Osteomyelitis is an infectious bone disease caused by pathogens that spread through contiguous infection, surgery/trauma-induced direct infection, or bacteremia from hematogenous dissemination. Its incidence has been rising annually, with risk factors including diabetes, peripheral vascular disease, immunodeficiency, drug abuse, and iatrogenic implants (Masters et al., 2022). Among these, diabetes is the major driving factor. According to the International Diabetes Federation, 10%–20% of the over 550 million diabetic patients worldwide may develop diabetes-related osteomyelitis, with the foot being the most vulnerable area due to peripheral vascular disease, nerve damage, and increased foot pressure (Kremers et al., 2015; Sun et al., 2022; Wilson et al., 2019). Unlike common osteomyelitis, diabetic foot osteomyelitis (DFO) is often accompanied by blood sugar control issues, peripheral vascular and nerve damage, resulting in suboptimal clinical outcomes. The amputation rate for hospitalized patients with DFO can be as high as 20% (Bokinni, 2024). Currently, there is no ideal clinical treatment plan for DFO patients.

## 2 Current management strategies and limitations of DFO

The current clinical treatment of DFO primarily includes the removal of infection/necrotic tissue, local/systemic application of antibiotics, and reconstruction of bone/soft tissue. However, due to impaired local blood circulation in DFO, maintaining adequate antibiotic concentration is difficult, leading to a recurrence rate exceeding 30% (Zhu et al., 2022). For bone infections, antibiotics-loaded PMMA bone cement is commonly used in clinical practice, maintaining high local antibiotic concentrations after debridement to control the infection. However, this treatment does not promote bone regeneration and relies on free bone grafting or Ilizarov bone transport for bone reconstruction. This not only greatly extends the treatment duration (Ren et al., 2023), but also increases the risk of pin tract infections and the likelihood of recurrence. To address both bone infection and bone defect in osteomyelitis, Han et al. reported the application of the Masquelet-induced membrane technique in the treatment of bone infection, promoting bone regeneration and reducing infection recurrence (Han et al., 2017). Ren et al. employed a stepwise debridement–reconstruction–docking (DRD) strategy, which reduced the rates of osteomyelitis recurrence and infected nonunion. However, this approach still involves multiple surgical procedures, prolonged treatment duration, and high costs for some patients (Ren et al., 2023). However, it still faces the practical problems of requiring multiple surgeries, long treatment cycles, and high costs. Therefore, there is an urgent need to develop a systematic, precise, and safe treatment strategy. Understanding the characteristic pathological microenvironment of DFO is a prerequisite for achieving this goal.

## 3 Microenvironment and key targets of DFO

**FIGURE 1:**
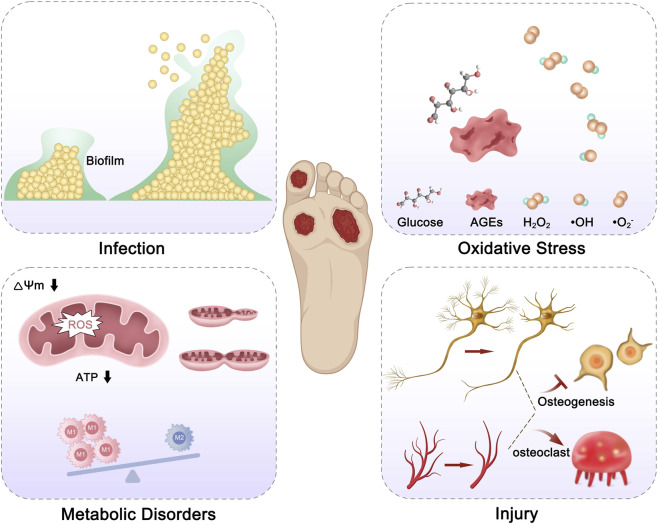
Microenvironment of DFO Infection, oxidative stress, metabolic disorders, and tissue injury jointly contribute to DFO progression. Abbreviations: ROS, reactive oxygen species; AGEs, advanced glycation end products; ΔΨm, mitochondrial membrane potential.

The microenvironment of DFO is characterized by several key features: (1) Hyperglycemia provides nutrients for pathogenic bacteria, leading to persistent and recurrent infections; (2) The infection site contains a large number of inflammatory cytokines, reactive oxygen species (ROS), and advanced glycation end-products (AGEs), which induce sustained oxidative stress and, in turn, trigger inflammatory responses (Bokinni, 2024); (3) The combined effect of ROS and AGEs disrupts the glycolysis/mitochondrial oxidative phosphorylation metabolic balance, leading to mitochondrial damage and macrophage polarization dysfunction, which further exacerbates the inflammatory response; (4) Microvascular and nerve lesions reduce bone formation, and under the combined action of infection and inflammation, an imbalance in osteoblast/osteoclast transformation occurs, leading to progressive bone destruction (Hofbauer et al., 2022) (Figure 1). From the above characteristics of the DFO microenvironment, three key targets can be identified: infection, inflammation, and bone defects, which are interdependent yet independent. Therefore, the key challenge lies in how to precisely, sequentially, and effectively address these issues. This review systematically summarizes the control of infection, inflammation, and promotion of bone tissue regeneration, and looks forward to potential directions for DFO treatment, aiming to provide practical and feasible insights for DFO management.

## 4 Multitarget combination antimicrobial strategies of DFO

**FIGURE 2:**
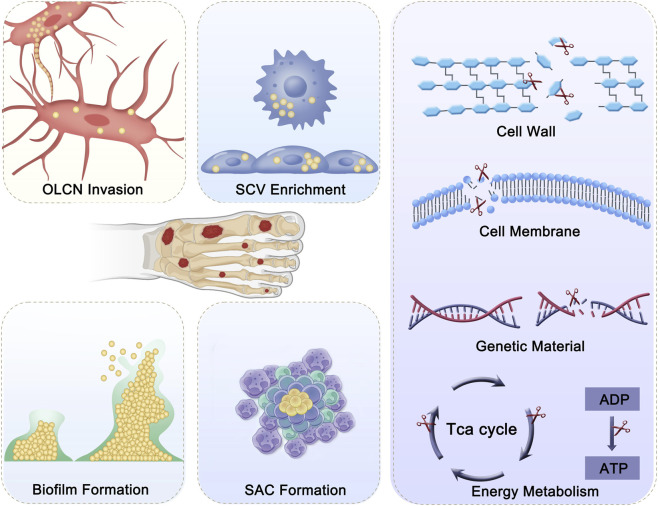
Bacterial persistence and antibacterial targets in DFO. Bacteria persist in DFO by invading the osteocyte lacuno–canalicular network (OLCN), forming biofilms, enriching small colony variants (SCVs), and developing staphylococcal abscess communities (SACs), which protect them from immune clearance and antibiotic penetration and promote chronic infection. Antibacterial strategies target bacterial survival by disrupting the cell wall and membrane, damaging genetic material, and inhibiting energy metabolism (e.g., the TCA cycle and ATP production), thereby reducing bacterial viability and persistence.

Antimicrobial treatment is the core step in DFO management. *Staphylococcus aureus* (*Staphylococcus aureus*) is the most common pathogen in DFO, with other common pathogens including *Coagulase-negative Staphylococci*, *Escherichia coli*, and *Pseudomonas aeruginosa* (Masters et al., 2022). Currently, the primary antimicrobial approach for DFO involves the extensive use of high-dose antibiotics locally and systemically after thorough debridement. However, treatment failure and recurrence rates remain high, primarily due to the following reasons: (1) After *S. aureus* invades bone tissue, it can colonize in the bone cell lacunae network (OLCN), where antibiotics cannot maintain effective concentrations due to the dense structure of the OLCN; (2) Impaired immune cell function allows bacteria that are phagocytosed to remain active inside the cells, facilitating systemic spread and promoting the accumulation of small colony variants (SCVs), which help evade host immune defenses; (3) The formation of bacterial biofilms provides a protective barrier for the bacteria; (4) *S. aureus* forms *Staphylococcal Abscess Communities* (SACs) after attacking immune cells, limiting the access of immune cells and antibiotics (Qin et al., 2024). In addition to antibiotics, commonly used antimicrobial agents in osteomyelitis treatment include antimicrobial peptides, antimicrobial enzymes, cationic polymers, metal-based antimicrobial agents, and natural extracts. These mainly act on bacterial cell walls, cell membranes, genetic material, and energy metabolism (Figure 2). Based on the above materials and antimicrobial targets, several research teams have recently reported multitarget antimicrobial strategies for bone infection treatment. For example, Professor Wei Chen’s team (Fan et al., 2025) synthesized bioactive borosilicate glass (BSG), which synergistically enhances the antibacterial activity of gentamicin, disrupting biofilm integrity, cell walls, and cell membranes. Additionally, Professor Shuilin Wu and Xiangmei Liu’s team (Li et al., 2020) developed a photothermal-active hydrogel, which, under the synergy of photothermal therapy (PTT) and positive ions, effectively clears biofilms and kills bacteria within bone. Experimental studies have shown that multitarget antimicrobial strategies can effectively eliminate primary bone infections. However, a significant challenge remains in preventing recurrence due to the immune-suppressive state in the body. Further research is needed to effectively prevent reinfection. In addition, ongoing explorations of bacteriophage-based therapies for antibiotic-resistant bone infections offer a feasible alternative strategy for infection control. Yao et al. (2025) reported a bacteriophage-based immune labeling strategy that enhances bacterial recognition and clearance by macrophages through mannose-mediated targeting and Mn^2+^-driven immunomodulation, thereby linking phage therapy with host innate and adaptive immune activation (Yao et al., 2025). Overall, multitarget antimicrobial strategies improve infection control in DFO by addressing multiple bacterial survival mechanisms; future advances will likely depend on integrating antimicrobial, immunomodulatory, and microenvironment-regulating approaches to achieve durable infection eradication and reduce recurrence.

## 5 Anti-inflammatory systems based on TCM-derived bioactive molecules

In the hyperglycemic microenvironment of DFO, cells are unable to fully utilize glucose (Glu), which primarily enters the glycolytic pathway. To maintain ATP levels within the cells, the production of fragmented and dysfunctional mitochondria increases, weakening mitochondrial oxidative phosphorylation and the mitochondrial respiratory chain. This leads to mitochondrial membrane potential depolarization, electron transport blockage, and damage to mitochondrial structure and function, further reducing antioxidant capacity and exacerbating oxidative stress. Persistent oxidative stress generates ROS and AGEs, triggering sustained inflammatory responses (Wang et al., 2024). Additionally, abnormal glycolysis disrupts the metabolic and functional plasticity of macrophages, delaying the transition of macrophages to the M2 phenotype. This results in the secretion of more pro-inflammatory and chemotactic factors, leading to the generation of a predominance of macrophage polarization toward the M1 phenotype, further intensifying the inflammatory response (Ginhoux et al., 2016). Therefore, reversing the sustained oxidative stress and inflammation is critical for accelerating the healing of DFO. Recent studies have highlighted that molecules from traditional Chinese medicine (TCM), such as Shikonin (Guo et al., 2023), Salvianolic acid B(Sal B) (Zhang et al., 2025), Quercetin (Kumar et al., 2024), Tannic acid (Fang et al., 2025), Ferulic acid (Li et al., 2025b), EGCG (Gong et al., 2025), Salidroside (Zheng et al., 2025), Myricetin (Ansari et al., 2025), and Myricitrin (Ahangarpour et al., 2018), can participate in various physiological processes, including inflammation, glycolysis, cell cycle, and immune regulation through multiple signaling pathways. These compounds have shown promising therapeutic effects in diseases such as infections, inflammatory bowel disease, chronic wounds, and arthritis. Zhang et al. reported a system using Sal B to improve diabetic skin wound healing through Pink1/Parkin-mediated mitophagy. This mechanism reduces oxidative stress, enhances mitochondrial function, and mitigates inflammation. In a diabetic mouse model, Sal B promoted wound healing, angiogenesis, and reduced inflammation. It upregulated Parkin and Pink1, improving mitochondrial health (Zhang et al., 2025). Gong et al. achieved wound healing in diabetic foot ulcers (DFU) using a metal–polyphenol nanocomposite hybrid hydrogel. The system integrates EGCG (Epigallocatechin gallate) and Fe^3+^ within the hydrogel to enhance its antioxidant, anti-inflammatory, and antibacterial properties. The hydrogel is designed to release embedded metal nanoparticles and EGCG in response to the acidic environment of DFU, improving the local microenvironment. In an *in vitro* RAW264.7 macrophage model and bacterial culture assays, the system demonstrated strong ROS scavenging activity, improved wound healing, and inhibited bacterial growth. Advantages include its multifunctionality in promoting angiogenesis, osteogenesis, and neurogenesis while addressing oxidative stress (Gong et al., 2025). Kumar et al. (2024) reported a PLGA/gelatin nanofiber patch incorporating CaCO3/SiO2 nanocomposite and quercetin to accelerate diabetic wound healing. The patch demonstrated antibacterial properties, promoted cell proliferation, and improved vascularization in in vitro and *in vivo* models. It showed promising results in enhancing wound healing, but challenges remain in scaling production and drug incorporation. The patch’s biocompatibility and efficacy suggest it could be a viable option for treating diabetic ulcers (Kumar et al., 2024).The active components in traditional Chinese medicine are effective in controlling inflammation and oxidative stress, and can promote DFO healing by targeting key physiological processes. However, there is a significant gap in their clinical application, and developing related materials is a feasible direction for future research. However, the current evidence for these approaches is mainly derived from *in vitro* studies, animal models, or early translational research, and their clinical application will require further validation through well-designed clinical trials.

## 6 Vascular–neural–bone integrated regeneration strategies

Bone defect reconstruction is a key component in the treatment of DFO. Bone repair is not an isolated process, but involves cross-talk with blood vessels and nerves, alongside a strong immune response. Within the skeleton, blood vessels and nerves (including sensory and sympathetic nerves) coexist and are widely distributed (Tao et al., 2023). The vascular network provides oxygen and nutrients to the skeletal system and secretes and delivers growth factors that regulate bone and nerve regeneration. Sensory nerves promote vascular regeneration and osteogenic differentiation by secreting neurotrophic factors and neuropeptides (Zhang et al., 2016). Motor neurons, including adrenergic and cholinergic nerves, have a negative and positive regulatory effect on bone formation, respectively. Furthermore, neuropeptides can promote macrophage M2 polarization and secrete various growth factors to facilitate vascular, nerve, and bone formation (Tao et al., 2023). Under the combined effects of infection, oxidative stress, and inflammation, pathological bone destruction occurs locally in DFO infections. Meanwhile, the peripheral vascular and nerve lesions associated with DFO lead to impaired bone regeneration capacity. Therefore, simple osteogenic strategies are ineffective in accelerating bone regeneration in DFO. In recent years, novel bone tissue engineering materials have achieved bone defect repair in complex environments through dynamic coordination among osteocytes, osteogenic factors, and biomaterials. For example, Professor Bobin Mi, Guohui Liu, and Qian Feng’s team (Zha et al., 2024) developed a HA-based hydrogel that clears bacteria in the early stages of infected bone defects and continuously releases various active metal ions to participate in angiogenesis, neurogenesis, and osteogenesis. Professor Jinghui Huang’s team (Li et al., 2025a) integrated extracellular vesicles derived from deer antler precursor cells with HAMA hydrogel, coordinating vascular, neural, and immune-related signals to accelerate bone growth. These studies demonstrate that bone tissue engineering materials with multiple active functions can accelerate bone regeneration by regulating the cross-talk between vascular, neural, and immune cells, showing great potential for application in DFO patients with vascular and nerve lesions.

## 7 Conclusion and outlook

The therapeutic ceiling in DFO is largely determined by a hostile and heterogeneous local microenvironment. Several constraints are recurrent. Glucose-rich niches can sustain bacterial persistence. Excess cytokines, ROS, and AGEs impose a sustained oxi-inflammatory load that entrenches chronic inflammation. Metabolic disruption further impairs immune competence. In parallel, microvascular insufficiency and neuropathic injury suppress osteogenesis and accelerate progressive bone loss. Together, these factors map onto three tightly coupled targets: infection, inflammation, and bone defects. Importantly, their relative dominance shifts across disease stages rather than remaining fixed. DFO management should therefore be tailored to the leading barrier at a given time point (e.g., bacterial burden, unresolved inflammation, or regenerative failure). It should also be tailored to patient-level determinants, including perfusion status, neuropathy severity, and recurrence risk. A stage-adaptive sequence is needed, and it should avoid “one-size-fits-all” escalation.

To date, most approaches have attempted to address these targets in parallel but in practice often in isolation, typically prioritizing infection control and deferring reconstruction. This can create a timing mismatch with an evolving microenvironment, which in turn prolongs treatment courses and increases the likelihood of relapse. By contrast, emerging biomaterials increasingly offer multifunctionality. They can combine antimicrobial effects with anti-oxidative/anti-inflammatory regulation and pro-regenerative support, enabling coordinated control of key bottlenecks within a unified therapeutic logic. Looking ahead, integrated platforms are well positioned to deliver tailored, microenvironment-responsive interventions by unifying antibacterial, immunoregulatory, and regenerative functions within a modular system. Using physico-chemical tuning and programmable payload delivery, these platforms can be engineered to adjust dose, timing, and spatial distribution in response to local cues (e.g., glucose, ROS, acidity, or enzymatic activity). This strategy aligns therapy with the patient’s evolving microenvironment. It also supports precision DFO care by reducing overtreatment, limiting collateral tissue injury, and improving the durability of infection control while rebuilding the neurovascular-bone unit.
